# *ABCA1* and *ABCG1* DNA methylation in epicardial adipose tissue of patients with coronary artery disease

**DOI:** 10.1186/s12872-021-02379-7

**Published:** 2021-11-27

**Authors:** Valentina V. Miroshnikova, Alexandra A. Panteleeva, Irina A. Pobozheva, Natalia D. Razgildina, Ekaterina A. Polyakova, Anton V. Markov, Olga D. Belyaeva, Olga A. Berkovich, Elena I. Baranova, Maria S. Nazarenko, Valery P. Puzyrev, Sofya N. Pchelina

**Affiliations:** 1 Petersburg Nuclear Physics Institute Named By B.P. Konstantinov of National Research Center “Kurchatov Institute”, Gatchina, Russian Federation; 2 Pavlov First Saint Petersburg State Medical University, St.-Petersburg, Russian Federation; 3 National Research Centre “Kurchatov Institute”, Moscow, Russia; 4 Laboratory of Population Genetics, Research Institute of Medical Genetics, Tomsk, Russian Federation

**Keywords:** Coronary artery disease, ABCA1 and ABCG1 transporters, Epicardial adipose tissue, DNA methylation

## Abstract

**Background:**

Recent studies have focused on the potential role of epicardial adipose tissue (EAT) in the development of coronary artery disease (CAD). ABCA1 and ABCG1 transporters regulate cell cholesterol content and reverse cholesterol transport. We aimed to determine whether DNA methylation and mRNA levels of the *ABCA1* and *ABCG1* genes in EAT and subcutaneous adipose tissue (SAT) were associated with CAD.

**Methods:**

Paired EAT and SAT samples were collected from 82 patients undergoing elective cardiac surgery either for coronary artery bypass grafting (CAD group, N = 66) or valve surgery (NCAD group, N = 16). *ABCA1* and *ABCG1* mRNA levels in EAT and SAT samples were analyzed using real time polymerase chain reaction, ABCA1 protein levels in EAT samples were assessed by western blotting. *ABCA1* and *ABCG1* DNA methylation analysis was performed in 24 samples from the CAD group and 9 samples from the NCAD group via pyrosequencing.

**Results:**

DNA methylation levels in the *ABCA1* promoter and *ABCG1* cg27243685 and cg06500161 CpG sites were higher in EAT samples from patients with CAD compared with NCAD (21.92% vs 10.81%, p = 0.003; 71.51% vs 68.42%, p = 0.024; 46.11% vs 37.79%, p = 0.016, respectively). In patients with CAD, *ABCA1* and *ABCG1* DNA methylation levels were higher in EAT than in SAT samples (p < 0.05). *ABCA1* mRNA levels in EAT samples were reduced in the subgroup of patients with CAD and concomitant carotid artery disease or peripheral artery disease compared with the NCAD group (p = 0.024). ABCA1 protein levels in EAT samples tended to be lower in CAD patients than in the NCAD group (p = 0.053). DNA methylation levels at the *ABCG1* cg27243685 site positively correlated with plasma triglyceride concentration (r = 0.510, p = 0.008), body mass index (r = 0.556, p = 0.013) and waist-to-hip ratio (r = 0.504, p = 0.012) in SAT samples.

**Conclusion:**

CAD is associated with *ABCA1* and *ABCG1* DNA hypermethylation in EAT. CAD with concomitant carotid artery disease or peripheral artery disease is accompanied by decreased *ABCA1* gene expression in EAT. DNA methylation levels at the *ABCG1* cg27243685 locus in SAT are associated with hypertriglyceridemia and obesity.

**Supplementary Information:**

The online version contains supplementary material available at 10.1186/s12872-021-02379-7.

## Background

Obesity has been linked with cardiometabolic disorders such as insulin resistance, atherogenic dyslipidemia and hypertension as well as cardiovascular diseases [1]. Total body adiposity correlates with the amount of epicardial adipose tissue (EAT), a specific visceral fat accumulation around the myocardium in the proximity to the coronary arteries [2]. In recent years, an association between EAT thickness and coronary artery disease (CAD) has been reported, and EAT has been proposed to play a role in the pathogenesis of CAD [3–6]. Adipose tissue is considered to be an endocrine organ producing adipocytokines that might modulate atherosclerosis progression [7–9]. Several lines of evidence suggest that adipose tissue reverse cholesterol transport impairment leads to adipocyte cholesterol imbalance and dysfunction [10–12]. It is worth noting that reverse cholesterol transport could be disrupted in EAT of CAD patients [9].

Cholesterol efflux from adipocytes is mediated by the cholesterol transport proteins ATP-binding cassette A1 (ABCA1) and ATP-binding cassette G1 (ABCG1). ABCA1 regulates the formation of nascent high-density lipoprotein (HDL) via cholesterol efflux to lipid free apolipoprotein A-1, whereas ABCG1 mediates cholesterol transport to the HDL fraction [13]. Adipocyte Abca1 deficiency is associated with HDL reduction in animal models, suggesting that adipose tissue contributes to HDL formation as well [14, 15]. *ABCA1* and *ABCG1* gene expression in subcutaneous and visceral adipose tissue has been shown to be dysregulated in obesity and during metabolic syndrome development [16–19]. However, whether ABCA1 and ABCG1 activity plays a role in EAT of CAD patients remains unknown.

Recent findings suggest that DNA methylation changes are related to the development and progression of a group of human diseases including atherosclerosis. Previously, we and others demonstrated differences in genome-wide DNA methylation patterns between atherosclerotic plaques and normal arteries [20–22]. Differential methylation within the *ABCA1* and *ABCG1* regulatory regions in leukocytes has been previously shown to contribute to the interindividual variability in plasma HDL concentrations and was associated with atherosclerosis-related diseases [23–28]. Taken together, the data indicate a key role for DNA methylation of the *ABCA1* and *ABCG1* genes in the development of diseases associated with impaired lipid metabolism [28]. We hypothesized that *ABCA1* and *ABCG1* DNA methylation patterns may be altered in EAT of CAD patients.

The aim of this study was to investigate the *ABCA1* and *ABCG1* DNA methylation status and gene expression in paired samples of EAT and subcutaneous adipose tissue (SAT) from patients with CAD.

## Methods

### Patients

A total of 82 patients diagnosed with CAD and heart valve disease who underwent cardiac surgery from September 2016 to April 2017 were enrolled in this study.

Coronary angiography was performed in all participants to identify coronary atherosclerosis, and patients were divided into two groups: CAD patients (n = 66) or non-CAD subjects (NCAD; n = 16) as a control group. Inclusion to the CAD group was determined via coronary angiography and included patients with 1-2-3-vessel coronary obstruction, who underwent further coronary bypass grafting surgery. The NCAD group included patients undergoing open-heart surgery for valvular replacement without stenosis in the coronary artery lumen. Clinical characteristics, including demographic data, adiposity parameters [body mass index (BMI), waist circumference, waist-to-hip ratio], lipid profiles, smoking and medical history were obtained from the hospital records. The main clinical characteristics of the studied groups are presented in Table 1. The exclusion criteria were as follows: cancer, chronic obstructive pulmonary disease, liver or renal failure, connective tissue diseases, acute rheumatic fever, infective endocarditis, hypo/hyperthyroidism, brain diseases, alcohol or drug abuse and acute cerebrovascular accident. EAT thickness was measured by echocardiography using the GE VIVID 7 Dimension cardiovascular ultrasound system, in front of the right ventricle wall from the parasternal long axis view.

**Table 1:** Clinical characteristics of the studied groups

Variables	CAD patients N = 66	NCAD patients (Control group) N = 16	*P* value
Age, years	62 (38–78)	64 (29–75)	0.727
Age of CAD, years	53 (37–73)	–	–
Male/female	49/17	8/8	> 0.05
BMI, kg/m^2^	27.9 (21.1–41)	23.9 (20.9–32.9)	0.029
Waist circumference, cm	Males	Males	
	98.5 (81–125)	84 (69–104)	0.001
	Females	Females	
	91 (79–113)	92 (58–103)	0.888
Waist-to-hip ratio	Males: 0.96 ± 0.04	Males: 0.89 ± 0.04	0.024
	Females: 0.82 ± 0.06	Females: 0.83 ± 0.11	0.931
EAT thickness, mm	6.49 ± 2.52	5.33 ± 1.93	0.106
**Smoking**			
Never smoked	29 (44%)	14 (88%)	0.003
Ex-smokers	16 (24%)	1 (6%)	0.100
Smoking during the time of the study	12 (18%)	0 (0%)	0.060
Smoking status is unknown	9 (14%)	1 (6%)	–
**Features of the diagnosis:**			
Patients with one-vessel coronary artery disease, N (%)	7 (10.1%)	–	–
Patients with two-vessel coronary artery disease, N (%)	16 (23.2%)	–	–
Patients with three-vessel coronary artery disease, N (%)	46 (66.7%)	–	–
Patients with CAD and established concomitant carotid artery disease or peripheral artery disease, N (%)	8 (12%)	–	–
**Biochemical parameters**			
Triglyceride, mmol/L	1.47 (0.35–5.09)	1.07 (0.40–3.82)	0.042
Total cholesterol, mmol/L	4.85 ± 1.47	4.57 ± 1.02	0.592
HDL-cholesterol, mmol/L	1.21 (0.70–2.35)	1.20 (0.50–1.82)	0.369
LDL-cholesterol, mmol/L	2.59 ± 1.34	2.84 ± 0.53	0.339
Fasting glucose, mmol/L	5.55 (4.50–8.80)	5.25 (4.30–8.70)	0.083

The study protocol is in accordance with the Declaration of Helsinki and was approved by the local ethics committee of Pavlov First Saint-Petersburg State Medical University, Saint-Petersburg, Russian Federation. Written informed consent was given by each participant.

### Adipose tissue samples

Paired EAT and SAT samples were obtained during cardiac surgery from approximately the same location in all patients. EAT was obtained near the right coronary artery ostium and SAT from the area of chest incision. The tissue samples (average 0.1 g) were separated from any attached connective tissue and blood vessels and were stored at -80ºC for further analysis.

### RNA isolation and quantitative real-time PCR

Total cellular RNA was extracted using RNeasy Mini columns (Qiagen, Germany) following the manufacturer’s protocol. The concentration and purity of the extracted RNA were assessed by calculating the ratio of optical density at 260 and 280 nm (OD 260/280), and the integrity of RNA was reflected by the presence of 18S and 28S ribosomal bands in electrophoresis through 1% agarose gels. A total of 1 µg of RNA from each biopsy was reverse transcribed with RevertAid cDNA Synthesis Kit (Thermo Fisher Scientific, USA) according to the instruction manual. Quantitative real-time PCR analysis was conducted using the CFX96 Real-Time PCR Detection System (Bio-Rad, USA). Primers and probes for TaqMan RT-PCR of the *ABCA1* and *ABCG1* and the reference genes are listed in Additional file 1: Table S1. Each reaction contained 1 µL of cDNA, 0.5 µL of each primer (10 µmol/L), 0.8 µL of TaqMan probes (10 µmol/L), 8 µL of deionized water and 10 µL of Master Mix (AlcorBio, Russia). Amplification was performed as follows: 3 min at 95 °C, then 45 cycles of 15 s at 95 °C, 15 s at 58 °C, 15 s at 72 °C. Threshold cycle (Ct) values were obtained and relative gene expression was normalized to two reference genes (*RPLP0* and *ACTB*), as previously described [19].

### DNA methylation analysis

DNA was isolated from ~ 50 mg of EAT and SAT samples using standard proteinase K digestion and phenol–chloroform extraction method. DNA quality and quantity were analyzed using the NanoDrop 8000 UV–Vis spectrophotometer (Thermo Scientific; Waltham, MA). Maximum 24 samples from the CAD group and 9 samples from the NCAD group for each type of adipose tissue were available for methylation analysis according to sufficient amount of material and DNA quality tests. Subsequently, 500 ng of genomic DNA with a 260/280 nm extinction ratio > 1.8 were treated with bisulfite using the EZ DNA Methylation Kit (Zymo Research Inc., USA), according to the manufacturer’s instructions. The DNA methylation assay for locus 1 (cg27243685) of the *ABCG1* gene was designed with the MethPrimer Software [29]. Analysis of the remaining CpG sites was performed according to the previous studies [30, 31]. The genomic location and primer sequences are presented in Table 2. For PCR, 40–50 ng of bisulfite-converted DNA was amplified using 2 U Hot Start Taq DNA polymerase (AlcorBio, Russia) and 0.2 μM forward and biotinylated reverse primers in a 50-μl reaction volume including 0.2 mM dNTPs and 2 mM MgCl_2_. PCR conditions were: 95 °C for 5 min followed by 40 cycles of 95 °C for 30 s, 58 °C for 30 s, 72 °C for 30 s and a final elongation step at 72 °C for 7 min. For DNA methylation levels, the percentage of 5-methylcytosine at individual CpG sites of the *ABCA1* and *ABCG1* was assessed by pyrosequencing using PyroMark Q24 (Qiagen Inc.) according to the manufacturer’s instructions. Biotin-labeled, single-stranded amplicons were retrieved and subjected to pyrosequencing using 0.3 μM sequencing primer, according to the manufacturer’s protocol. The percentage of methylation for each of the CpG sites within the target sequence was calculated using PyroQ CpG Software (Qiagen Inc.). Non-CpG cytosine residues were used as built-in controls to verify bisulfite conversion. Each sample was tested in two replicates and their average was used in the statistical analysis.

**Table 2:** Genes and primers for pyrosequencing

Gene	Genomic position of CpG sites (GCRh37/hg19)	Primer sequence
*ABCA1*	chr9:107,690,762; chr9:107,690,770 (cg14019050); chr9:107,690,773; chr9:107,690,791; chr9:107,690,797	5′-AACAAATTCCACTAATACCCTTAACT-3′ 5′-biotin-GGGTGGAGGGTATAGTAGGT-3′ Seq 5′-AACAAATTCCACTAATACCCTTAACT-3′
*ABCG1* locus 1	chr21:43,642,336; chr21:43,642,354; chr21:43,642,367 (cg27243685)	5′-TGAGTTTAGGAGGTTAAGGAGAAATT-3′ 5′-biotin-CAAATAAACCAACAACAAAACAATAC-3′ Seq 5′-TGAGTTTAGGAGGTTAAGGA-3′
*ABCG1* locus 2	chr21:43,656,587 (cg06500161); chr21:43,656,590	5′-GTAAGGTTTGGGGTTATTTTAGTGG-3′ 5′-biotin-AAAACAAACCCTTAAATCTCTTTACAT-3′ Seq 5′-GAGATTAGGGTTTTTTTTAGATA-3′

### ABCA1 western blotting

Tissue was ground in liquid nitrogen using a mortar, and the powder was homogenized in ice-cold RIPA buffer containing 50 mMTris-HCl (pH 8.0), 150 mMNaCl, 1% Triton X-100, 0.5% sodium deoxycholate, 0.1% SDS and protease inhibitor cocktail (Roche, Switzerland). The lysate was centrifuged at 14,000*g* for 15 min at 4 °C, and the fraction below the lipid portion on the top was carefully aspirated into a new tube. Protein concentrations were determined using a BCA protein assay (Pierce, USA). A mass of 5 µg protein per lane was separated using 8% SDS-PAGE gels. Proteins were transferred to PVDF membranes (Millipore, USA) and pre-incubated with 5% skim milk in PBS. The blots were incubated with polyclonal rabbit anti-ABCA1 (1:1000; ab7360, Abcam, United Kingdom) and polyclonal rabbit anti-β-actin (1:5000; ab8227, Abcam, United Kingdom) diluted in 1% skim milk in PBST (0.05% Tween 20) to prevent non-specific binding and followed by anti-rabbit HRP-conjugated secondary antibodies (1:3000; ab6721, Abcam, United Kingdom). Proteins were visualized using an enhanced chemiluminescence detection system (Amersham, United Kingdom).

### Statistical analysis

Normality of the data was tested using the Shapiro–Wilk test. Depending on the distribution, data are presented either as the mean ± SD or as the median and min–max range. Categorical variables are reported as frequencies and percentages (%). Mean values were compared using t-tests, whereas non-parametric data were compared using Mann–Whitney’s U-test (between the CAD and NCAD groups). Differences between DNA methylation and mRNA levels in EAT and SAT were assessed using Wilcoxon’s signed-rank test. Pairwise comparisons were Holm-Bonferroni corrected for four comparisons (two paired, two unpaired). Categorical variables were analyzed by Fisher's exact test. Spearman correlation testing was performed between methylation levels, mRNA and clinical and biochemical markers. Statistical analysis was performed using SPSS 17.0 software (SPSS Inc., Chicago, IL, USA) and in R statistical computing environment (the R Foundation, Vienna, Austria). Results with a p value < 0.05 were considered to be statistically significant.

## Results

In this study, we evaluated the association of *ABCA1* and *ABCG1* DNA methylation and gene expression in EAT and SAT with CAD along with CAD risk determinants: adiposity parameters (BMI, waist circumference, waist-to-hip ratio), lipid profiles and smoking status. The clinical characteristics of CAD patients and NCAD controls are shown in Table 1. Compared with the NCAD group, the CAD group was more likely to be overweight or obese, demonstrating slightly higher plasma triglyceride level, but without significant differences in the remaining lipid parameters.

The *ABCA1* promoter region and two loci near cg06500161 and cg27243685 CpG sites in *ABCG1* were selected for DNA methylation analysis because of their association with gene expression, lipid levels and cardiometabolic complications from several previous studies [23–28, 32–37]. The locations of the analyzed loci are shown in Additional file 2: Fig. S1. Methylation analysis included five CpG sites in the *ABCA1* promoter region (including cg14019050), as well as three and two CpG sites in introns 1 and 2 of the *ABCG1* gene, respectively (including the well-studied cg27243685 and cg06500161 sites) (Table 2). The differences for all CpG sites are provided in Additional file 3: Table S2 and Additional file 4: Table S3.

**Fig. 1:**
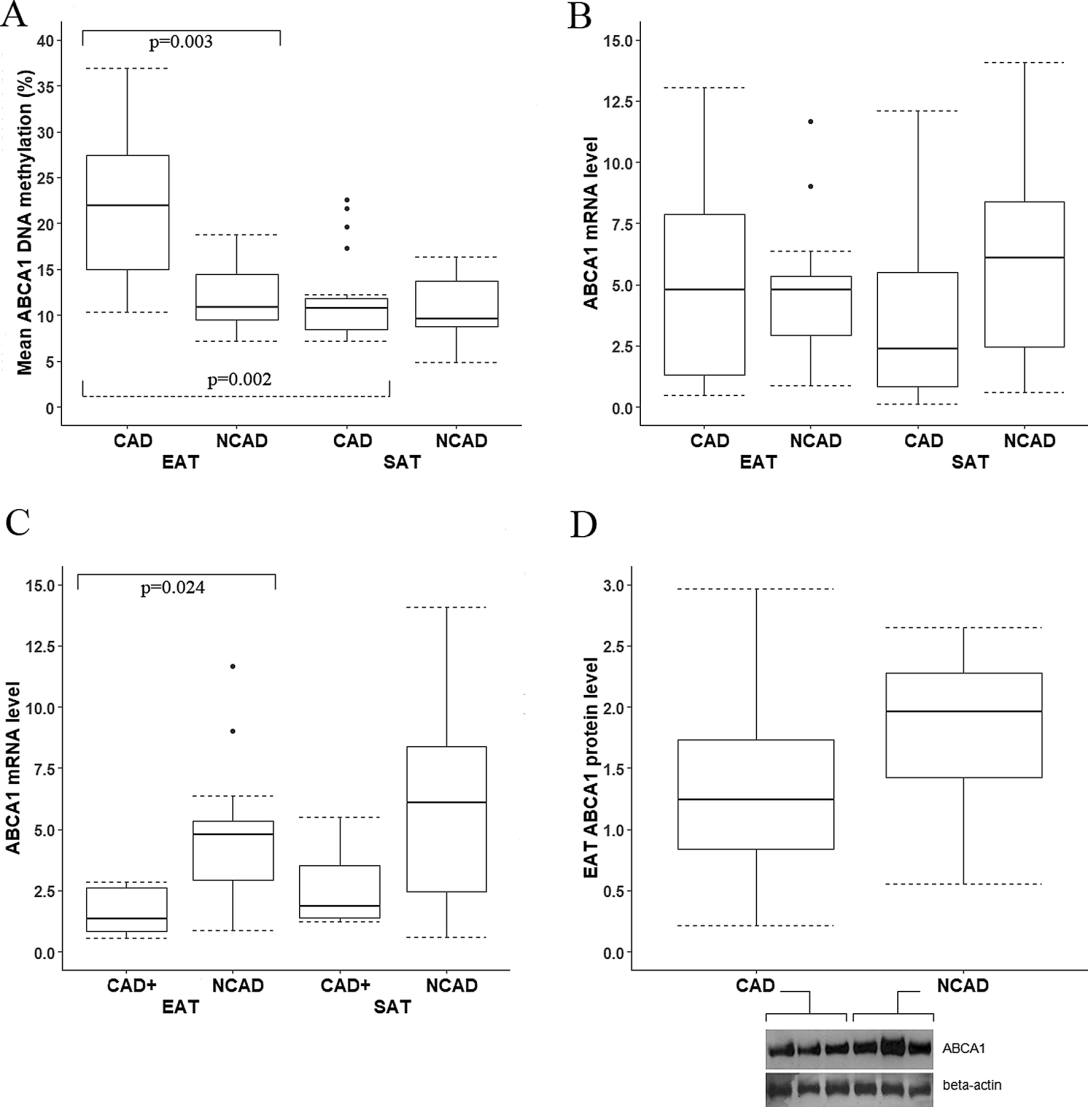
**A** Mean *ABCA1* DNA methylation levels in EAT and SAT; **B** *ABCA1* mRNA levels in EAT and SAT samples from the CAD and NCAD groups; **C** *ABCA1* mRNA levels in EAT and SAT samples from the subgroup of patients with CAD and concomitant carotid artery disease or peripheral artery disease compared to the NCAD group; **D** *ABCA1* protein levels in EAT samples from the CAD and NCAD groups and representative western blot. The origin bands of WB are presented in Additional file 5. *Note*: The CAD+ subgroup includes patients with CAD and concomitant carotid artery disease or peripheral artery disease

Hypermethylation at all the analyzed loci in EAT samples was associated with CAD. Indeed, DNA methylation levels at the *ABCA1* promoter region in EAT were 1.8-fold higher in patients with CAD compared with the NCAD group [21.92 (10.29–36.93) % vs 10.81 (7.12–18.74) %, p = 0.003; Fig. 1A]. Differences were significant for all five CpG sites when analyzed separately (Additional file 2: Table S2). *ABCA1* promoter methylation levels in SAT did not differ between the CAD and the NCAD groups (Fig. 1A; Additional file 3: Table S2).

**Fig. 2:**
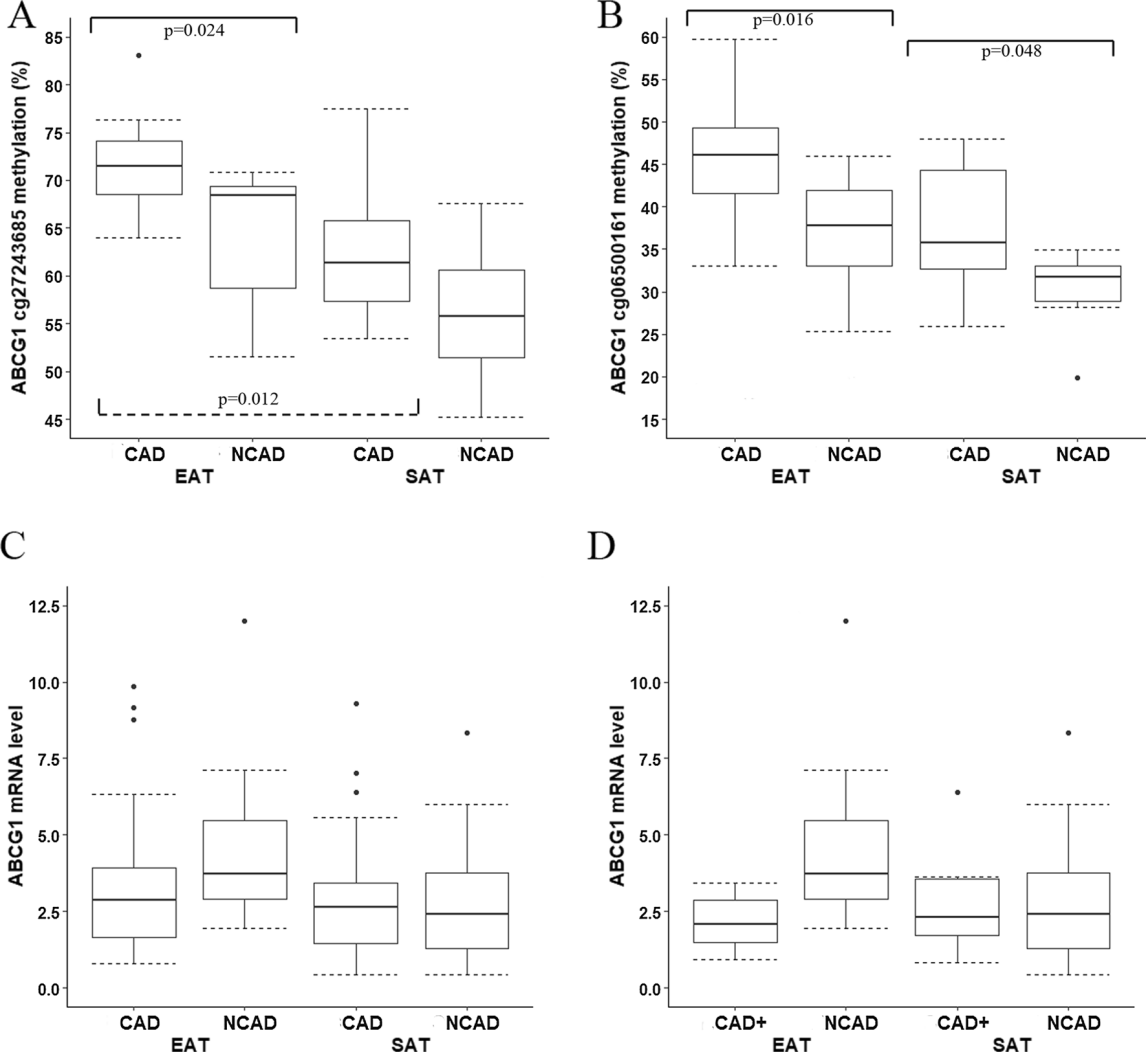
**A** *ABCG1* cg06500161 locus methylation levels in EAT and SAT; **B** *ABCG1* cg27243685 locus methylation levels in EAT and SAT; **C** *ABCG1* mRNA levels in EAT and SAT samples from the CAD and NCAD groups; **D** *ABCG1* mRNA levels in EAT and SAT samples from the subgroup of patients with CAD and concomitant carotid artery disease or peripheral artery disease compared to the NCAD group. *Note*: The CAD+ subgroup includes patients with CAD and concomitant carotid artery disease or peripheral artery disease

*ABCG1* methylation levels at both studied loci in EAT were higher in CAD patients compared with the NCAD group. Specifically, the mean *ABCG1* methylation levels at locus 1 in EAT were 71.15 (61.70–81.33) % in the CAD group and 65.66 (49.39–71.60) % in the NCAD group (p = 0.018). Differences were significant for all three CpG sites (Additional file 4: Table S3), including cg27243685 [71.51 (63.97–83.10) % vs 68.42 (51.56–70.88) %, p = 0.024; Fig. 2A]. The mean *ABCG1* methylation levels at locus 2 in EAT were 53.57 (42.17–63.70) % in the CAD group and 43.16 (33.78–54.32) % in the NCAD group (p = 0.004). Differences were significant for both CpG sites (Additional file 4: Table S3), including cg06500161 [46.11 (33.04–59.76) % vs 37.79 (25.33–46.00) %, p = 0.016; Fig. 2B].

Regarding DNA methylation at *ABCG1* locus 1 in SAT, there were no statistically significant differences for cg27243685, neither when analyzed separately, nor for mean DNA methylation levels (Additional file 4: Table S3). As for *ABCG1* locus 2, the mean DNA methylation levels were slightly higher in the CAD group [43.65 (33.38–68.73) % vs 39.70 (28.06–42.31) %, p = 0.020]. Mean methylation levels were also higher at each CpG site, including cg06500161 [36.17 (25.87–68.55) % vs 31.68 (19.82–34.93) %, p = 0.048; Fig. 2B].

ABCA1 protein levels in EAT had a tendency to be lower in the CAD group (Fig. 1D; p = 0.053) while mRNA levels did not differ (Fig. 1B). In addition, *ABCA1* mRNA levels in EAT were reduced in the subgroup of patients with CAD and concomitant carotid artery disease or peripheral artery disease (i.e. proven multifocal atherosclerosis) compared with the NCAD group (Fig. 1C). *ABCA1* mRNA levels in SAT also tended to be reduced in this subgroup, although the difference was not significant after adjustment for multiple comparisons. There were no differences in *ABCG1* mRNA levels in EAT and SAT between the CAD and NCAD groups (Fig. 2C). We did not analyze *ABCA1* and *ABCG1* DNA methylation levels in the subgroup of patients with CAD and concomitant carotid artery disease or peripheral artery disease separately, as only three samples from this subgroup were available for DNA methylation analysis. We did not find any significant association between DNA methylation level at each investigated site and corresponding gene’s mRNA levels which may be a consequence of limited sample size available for DNA methylation analysis.

*ABCA1* and *ABCG1* DNA methylation and gene expression levels were analyzed according to smoking history, considering smoking during the time of the study, as well as smoking for a long period but quitting smoking prior to the study (ex-smokers). *ABCA1* mRNA levels in SAT were reduced in combined group of smokers and ex-smokers [1.40 (0.16–8.42) vs 4.76 (0.13–14.07), p = 0.030; Additional file 6: Fig. S2] compared with patients who never smoked, indicating that mRNA levels did not increase after giving up smoking. DNA methylation levels were not associated with smoking status in this study.

**Fig. 3:**
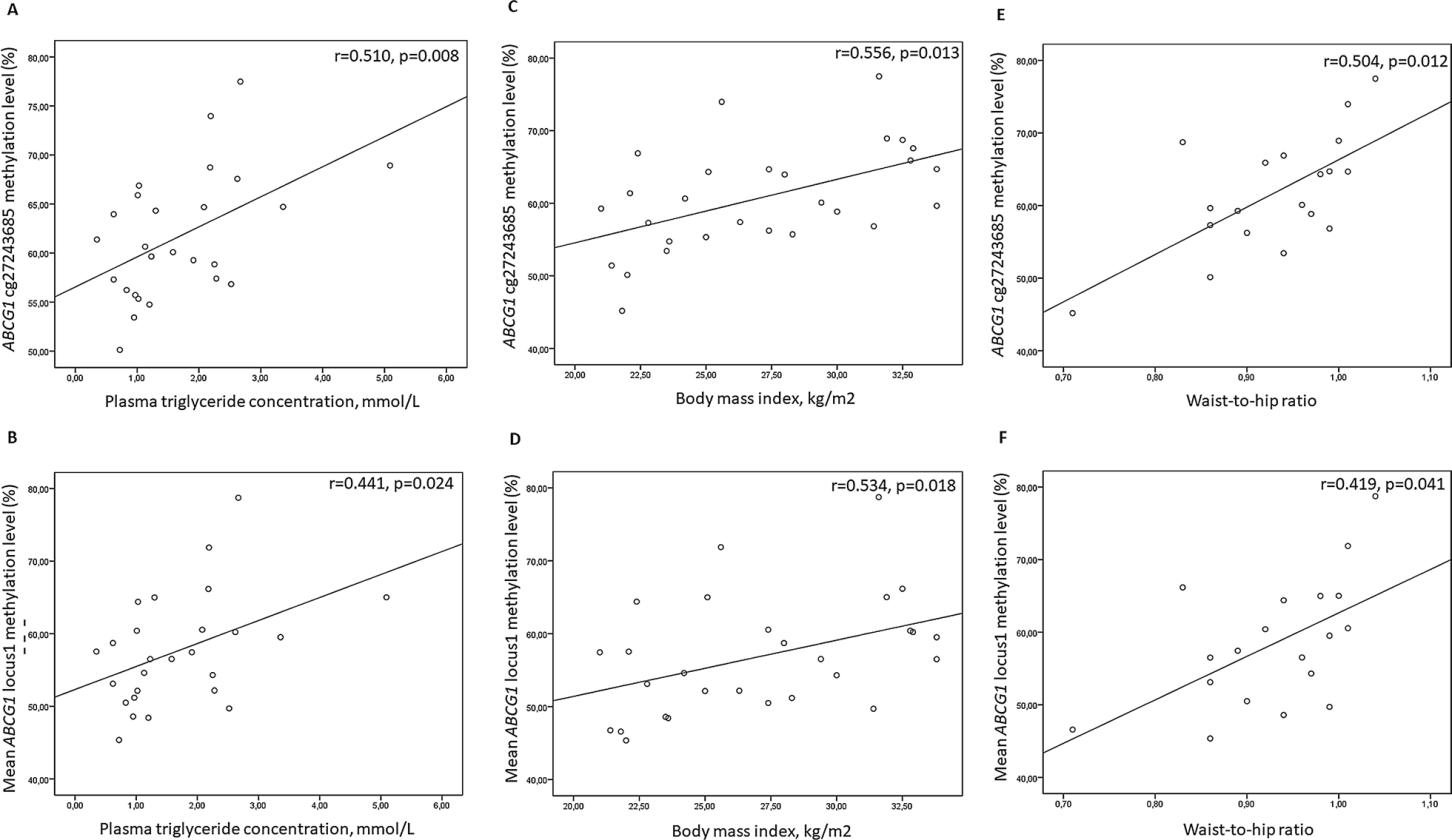
Spearman’s correlation analysis of *ABCG1* locus 1 methylation levels and (**A**, **B**) plasma triglyceride concentration; (**C**, **D**) body mass index; and (**E**, **F**) waist-to-hip ratio, if available

Correlation analysis of *ABCA1* and *ABCG1* DNA methylation and gene expression levels with plasma lipids and adiposity markers was carried out. DNA methylation levels of *ABCA1* in EAT and SAT, as well as *ABCG1* in EAT did not correlated with lipid concentrations or anthropometric parameters. As for *ABCG1* DNA methylation in SAT, there was a positive correlation between methylation levels at locus 1 and plasma triglycerides (r = 0.510, p = 0.008 for cg27243685; r = 0.441, p = 0.024 for mean DNA methylation levels at the locus), BMI (r = 0.556, p = 0.013 for cg27243685; r = 0.534, p = 0.018 for mean DNA methylation levels at the locus) and waist-to-hip ratio (r = 0.504, p = 0.012 for cg27243685; r = 0.419, p = 0.041 for mean DNA methylation levels at the locus) (Fig. 3). None of the measured parameters (*ABCA1* and *ABCG1* DNA methylation or mRNA levels) correlated with EAT thickness.

It should be noted that *ABCG1* locus 1 and locus 2 mean methylation levels positively correlated with each other (r = 0.629, p = 0.000; r = 0.577, p = 0.002, for EAT and SAT accordingly). The correlation analysis for all CpG sites within analyzed loci is shown in Additional file 7: Fig. S3. Methylation levels in CpGs sites within the same locus showed a strong positive correlation.

## Discussion

The study is the first to show that hypermethylation in the *ABCA1* promoter region and *ABCG1* CpG sites cg06500161 and cg27243685 in EAT is associated with CAD. We also demonstrated that *ABCA1* mRNA levels in EAT were reduced in CAD patients with concomitant carotid artery disease or peripheral artery disease, demonstrating a possible association of *ABCA1* expression in EAT with severe multifocal atherosclerosis. As *ABCA1* mRNA levels in EAT and SAT in the CAD group were very variable, it could not be excluded that some remaining CAD patients could also have asymptomatic plaques in other vascular areas. In addition to revealing differences in *ABCA1* mRNA levels and epigenetic changes in EAT between CAD patients and NCAD patients, we also assessed ABCA1 protein levels in EAT samples from both groups. *ABCA1* protein levels in EAT tended to be decreased in the CAD group compared with NCAD patients. These data indicate that the process of reverse cholesterol transport may be impaired in EAT of CAD patients. It has previously been demonstrated that reverse cholesterol transport from EAT in CAD patients can be disrupted also due to decreased secretion of apolipoprotein A1, a known ligand for ABCA1 [9]. This suggests that normal EAT is active for reverse cholesterol transport, and hypermethylation of key cholesterol transport genes, including *ABCA1* and *ABCG1*, may play a role in coronary atherosclerosis.

The majority of previous studies have investigated DNA methylation levels within *ABCA1* and *ABCG1* only in blood leukocytes. These studies have shown that DNA methylation of *ABCA1* and *ABCG1* was associated with a significant increase in the risk of atherosclerosis development and cardiovascular complications [23–28, 30, 38]. DNA methylation in the *ABCA1* promoter region is associated with prior history of CAD in patients with familial hypercholesterolemia [30]. Blood DNA methylation levels in *ABCG1* (cg06500161) are higher in subjects with previous hospitalized myocardial infarction compared with healthy controls [28]. *ABCG1* hypermethylation at the cg02494239 is associated with carotid intima media thickness and ischemic stroke [27]. Atherosclerosis is one of the primary causes of ischemic stroke and myocardial infarction and is characterized by the formation of atherosclerotic lesions, and the intima media thickness of the carotid artery is a subclinical stage of atherosclerosis [39]. Therefore, DNA methylation of *ABCA1* and *ABCG1* could be involved in atherosclerosis development.

The most probable mechanism underlying how *ABCA1* and *ABCG1* DNA methylation could contributes to the progression of atherosclerotic plaques could involve reduced gene expression and subsequent reduction of macrophage cholesterol efflux [13, 24, 40–42]. In previous studies, *ABCG1* has been shown to be hypermethylated and downregulated in blood monocytes/macrophages of CAD patients [24, 41, 43]. Furthermore, *ABCA1* mRNA levels are upregulated in macrophages, while protein levels are decreased [44, 45]. As adipose tissue is a complex tissue composed of different cell subsets, including macrophages, it may be hypothesized that EAT macrophages are critical for atherosclerotic plaques formation in the nearby coronary artery pool. It is important to mention that adiposity is associated with macrophage infiltration in adipose tissues, and it has been shown that *ABCG1* DNA methylation and gene expression could be affected by obesity [18, 46, 47].

Previous studies have demonstrated an inverse correlation between blood *ABCA1* and *ABCG1* DNA methylation and HDL levels [26, 30, 36, 37]. In this study, there was no association between *ABCA1* and *ABCG1* DNA methylation in adipose tissue and plasma cholesterol or HDL levels. However, *ABCG1* cg27243685 methylation in SAT was associated with higher plasma triglyceride concentration and positively correlated with BMI and waist-to-hip ratio. Thus, *ABCG1* expression in SAT could be influenced by DNA methylation and associated with abdominal obesity. A recent study has also shown that the correlation between BMI and *ABCG1* gene expression in monocytes is partially mediated by DNA methylation [31]. Previous studies have demonstrated that methylation of *ABCG1* CpG sites cg06500161 and cg27243685 in the blood is positively associated with plasma triglyceride concentration and could be linked to obesity and metabolic syndrome [28, 33, 48–52]. One possible explanation is that ABCG1 could regulate the bioavailability and the subsequent activity of lipoprotein lipase (LPL), a rate-limiting enzyme that hydrolyzes circulating triglyceride-rich lipoproteins [53, 54]. Reduced LPL activity in SAT could result in increased the plasma triglyceride levels, thus explaining the positive correlation between *ABCG1* methylation and triglyceride levels observed in this study [54]. A previous in vitro study has demonstrated a reduction in macrophage *ABCG1* expression when higher triglyceride levels were present in the culture medium, indicating that hypertriglyceridemia can contribute to macrophage reverse cholesterol transport reduction in the vascular wall [49]. Existing data suggests that obesity-related hypertriglyceridemia is associated with CAD risk via *ABCG1* gene expression dysregulation [42, 55].

Interestingly, *ABCA1* mRNA levels in SAT were reduced in patients with a history of smoking (including smokers and ex-smokers) compared with patients who never smoked, indicating that mRNA levels did not increase after giving up smoking. These results are in accordance with a previous study, in which a similar relationship between smoking and *ABCA1* expression in blood monocytes was observed [56]. However, DNA methylation levels in the *ABCA1* and *ABCG1* loci has not been associated with smoking status. No significant association was observed between *ABCA1* DNA methylation levels in blood and tobacco smoking in another study [28].

Thus, *ABCA1* and *ABCG1* mRNA levels in adipose tissue from CAD patients are likely to be influenced by additional risk factors, such as smoking, age, diet and obesity [26], which can partially explain the lack of association between *ABCA1* and *ABCG1* DNA methylation levels and the corresponding mRNA levels in our study. Gene expression is influenced by many factors in addition to DNA methylation, and currently, there is no clear understanding of the levels of DNA methylation required for transcriptional inactivation of different genes: thus, DNA methylation levels only partially predict gene expression [57]. mRNA levels are affected by additional processes, including microRNA-mediated degradation [58, 59]. We did not assess microRNA levels in this study.

The present study demonstrated higher levels of *ABCA1* DNA methylation in EAT compared with SAT in CAD patients. These observed differences in methylation levels in EAT and SAT partly could partly be linked to adipose tissue types. EAT is a type of visceral adipose tissue, whereas SAT represents fat depot of different origin [60]. Moreover, for *ABCG1* we observed a slight increase in *ABCG1* DNA methylation in EAT but also in SAT (significant for cg06500161). It may be hypothesized that changes in *ABCG1* DNA methylation in adipose tissue may be related partly to obesity, as it discussed above. Additionally, this could be a result of statin treatment, as differences in DNA methylation were seen in both types of adipose tissues. Indeed, statin users have been shown to have higher methylation levels in *ABCG1* CpG sites cg06500161 and cg27243685 in blood [61]. However, *ABCA1* methylation is not known to be influenced by statins [61]. Thus, increased methylation levels of *ABCA1* in EAT compared with SAT in CAD patients could be due to principal fat depot differences. This phenomenon could be linked to the pro-inflammatory profile of EAT, highlighting the importance of reverse cholesterol transport in EAT and supporting the hypothesis of epicardial fat tissue involvement in the pathogenesis of CAD.

There are several limitations to this study. One limitation is limited sample sizes of the studied groups, especially the NCAD group. Difficulties of material collecting are connected to variable interindividual amount of EAT which restricts dissecting the necessary size of EAT fragment in all cases, especially in the NCAD patients when EAT thickness is quite small. This additionally explains the fact that there are no differences in EAT thickness between the CAD and NCAD groups in this study. Another limitation is that patients from the CAD group, who were prescribed statins, were slightly overweight. Besides this, they were predominantly male and included a large proportion of smokers. Clinical heterogeneity and additional environmental factors might influence the expression profiles. The listed limitations partly explain that there was no association of mRNA levels with CAD and no correlation between DNA methylation and mRNA levels was found in this study. However we identified a subgroup of CAD patients with reduced *ABCA1* mRNA levels in EAT who underwent additional examination and had a proven concomitant carotid artery disease or a peripheral artery disease. Gender differences of obesity degree could be mentioned as a limitation. Although obesity was equally distributed in both studied groups waist circumference was higher in men with CAD. This could be addressed to a general feature of the population: women at the age usual for CAD manifestation are obese compared to men. To the best of our knowledge, our study is the first to analyze DNA methylation of the *ABCA1* and *ABCG1* genes in EAT. DNA methylation of these genes in blood leukocytes was not shown to be different between men and women in the other studies [24, 30]. Thus, we suppose gender differences of our studied groups are not linked to revealed changes in DNA methylation.

## Conclusion

*ABCA1* and *ABCG1* DNA hypermethylation in EAT is associated with CAD. Multifocal atherosclerosis is accompanied by decreased *ABCA1* mRNA expression in EAT. *ABCG1* methylation in SAT could be linked to hypertriglyceridemia and obesity. Taken together, our results support the involvement of *ABCA1* and *ABCG1* methylation in EAT in CAD development.

## Supplementary Information

**Additional file 1: Table S1.** Primers and probes for TaqMan RT-PCR.

**Additional file 2: Fig. S1.** Proportional diagram of the structure of *ABCA1* (NM_005502.4) (**A**) and *ABCG1* (NM_207174.1) (**A**) genes and loci selected for DNA methylation analysis. The line represents the gene (left to right: 5′–3′). Solid rectangles represent exons. Because of the long length of *ABCA1* and *ABCG1* genes, exons distant from the analyzed regions are omitted. ABCA1, ATP-binding cassette A1 gene; ABCG1, ATP-binding cassette G1 gene.

**Additional file 3: Table S2.** *ABCA1* promoter DNA methylation levels (%) in EAT and SAT in studied groups.

**Additional file 4: Table S3.** *ABCG1* DNA methylation levels (%) in EAT and SAT in studied groups.

**Additional file 5.** The origin bands of WB.The origin bands of WB.

**Additional file 6: Fig. S2.** *ABCA1* and *ABCG1* mRNA levels in EAT and SAT in subgroups divided according to smoking status. **A** ABCA1 mRNA levels in EAT; **B** ABCA1 mRNA levels in SAT (p = 0.030); **C** ABCG1 mRNA levels in EAT; **D** ABCG1 mRNA levels in SAT.

**Additional file 7: Fig. S3.** Spearman’s correlation coefficients (r) between CpG site methylation levels within analyzed loci: **A** for ABCA1 in SAT; **B** for ABCA1 in EAT; **C** for ABCG1 in SAT; **D** for ABCG1 in EAT. Filled squares in the case of ABCG1 gene represent correlations in the same locus, unpainted—between two different loci. *p < 0,01; **p < 0,05; for rest cells p = 0.000.

## Data Availability

DNA methylation data (pyrosequencing files) are available in GitHub repository https://github.com/lynxf/lab_ABCG1_ABCA1_methylation. Additional data is available from the corresponding author on reasonable request.

## Abbreviations

ABCA1: ATP-binding cassette transporter A1
ABCG1: ATP-binding cassette transporter G1
BMI: Body mass index
CAD: Coronary artery disease
EAT: Epicardial adipose tissue
HDL: High density lipoproteins
LDL: Low density lipoproteins
NCAD group: Non-CAD group
SAT: Subcutaneous adipose tissue
