# Supplementary material for: ABCA1 and ABCG1 DNA methylation in epicardial adipose tissue of patients with coronary artery disease
Source: BMC Cardiovasc Disord. 2021 Nov 27;21:566. doi: 10.1186/s12872-021-02379-7 (PMC8627066; doi:10.1186/s12872-021-02379-7)
Supplement: Supplementary file 1 — Additional file 1: Table S1. Primers and probes for TaqMan RT-PCR. [file 12872_2021_2379_MOESM1_ESM.docx]

Table S1. Primers and probes for TaqMan RT-PCR.

| Gene | Primer / probe structure (5' - 3') | Reference sequence |
| --- | --- | --- |
| *ABCA1* | 5`-СTCCTGTGGTGTTTCTGGATG-3’  5’-CTTGACAACACTTAGGGCACAA-3’  5’(FAM)-AAGCCCGGCGGTTCTTGTGG -3’(RTQ1) | [NM_005502.4](http://www.ncbi.nlm.nih.gov/entrez/query.fcgi?db=Nucleotide&cmd=Search&doptcmdl=GenBank&term=NM_005502.4) |
| *ABCG1* | 5’-CACGTACCTACAGTGGATGT-3’  5’-GTCTAAGCCATAGATGGAGA-3’  5’(FAM)-CTATGTCAGGTATGGGTTCGAAG-3’(RTQ1) | [NM_016818.2](https://www.ncbi.nlm.nih.gov/nucleotide/NM_016818.2?report=genbank&log$=nucltop&blast_rank=9&RID=8EV342CM014) [NM_004915.3](https://www.ncbi.nlm.nih.gov/nucleotide/NM_004915.3?report=genbank&log$=nucltop&blast_rank=8&RID=8EV342CM014) [NM_207174.1](https://www.ncbi.nlm.nih.gov/nucleotide/NM_207174.1?report=genbank&log$=nucltop&blast_rank=7&RID=8EV342CM014) [NM_207627.1](https://www.ncbi.nlm.nih.gov/nucleotide/NM_207627.1?report=genbank&log$=nucltop&blast_rank=6&RID=8EV342CM014) [NM_207628.1](https://www.ncbi.nlm.nih.gov/nucleotide/NM_207628.1?report=genbank&log$=nucltop&blast_rank=5&RID=8EV342CM014) [NM_207629.1](https://www.ncbi.nlm.nih.gov/nucleotide/NM_207629.1?report=genbank&log$=nucltop&blast_rank=4&RID=8EV342CM014) |
| *ACTB* | 5’-CGTGCTGCTGACCGAGG-3’  5’-ACAGCCTGGATAGCAACGTACA-3’  5’(R6G)-CCAACCGCGAGAAGATGACCCAGAT-3’(BHQ1) | - [NM_001101.4](http://www.ncbi.nlm.nih.gov/entrez/query.fcgi?db=Nucleotide&cmd=Search&doptcmdl=GenBank&term=NM_001101.5) |
| *RPLPO* | 5’-GATCAGGGACATGTTGCTGG-3’  5’-GACTTCACATGGGGCAATGG-3’  5’(ROX)-CAATAAGGTGCCAGCTGCTGC-3’(RTQ2) | - [NM_001002.4](http://www.ncbi.nlm.nih.gov/entrez/query.fcgi?db=Nucleotide&cmd=Search&doptcmdl=GenBank&term=NM_001002.4) - [NM_053275.3](http://www.ncbi.nlm.nih.gov/entrez/query.fcgi?db=Nucleotide&cmd=Search&doptcmdl=GenBank&term=NM_053275.3) |
