# Supplementary figures and images for: ABCA1 and ABCG1 DNA methylation in epicardial adipose tissue of patients with coronary artery disease
Source: BMC Cardiovasc Disord. 2021 Nov 27;21:566. doi: 10.1186/s12872-021-02379-7 (PMC8627066; doi:10.1186/s12872-021-02379-7)

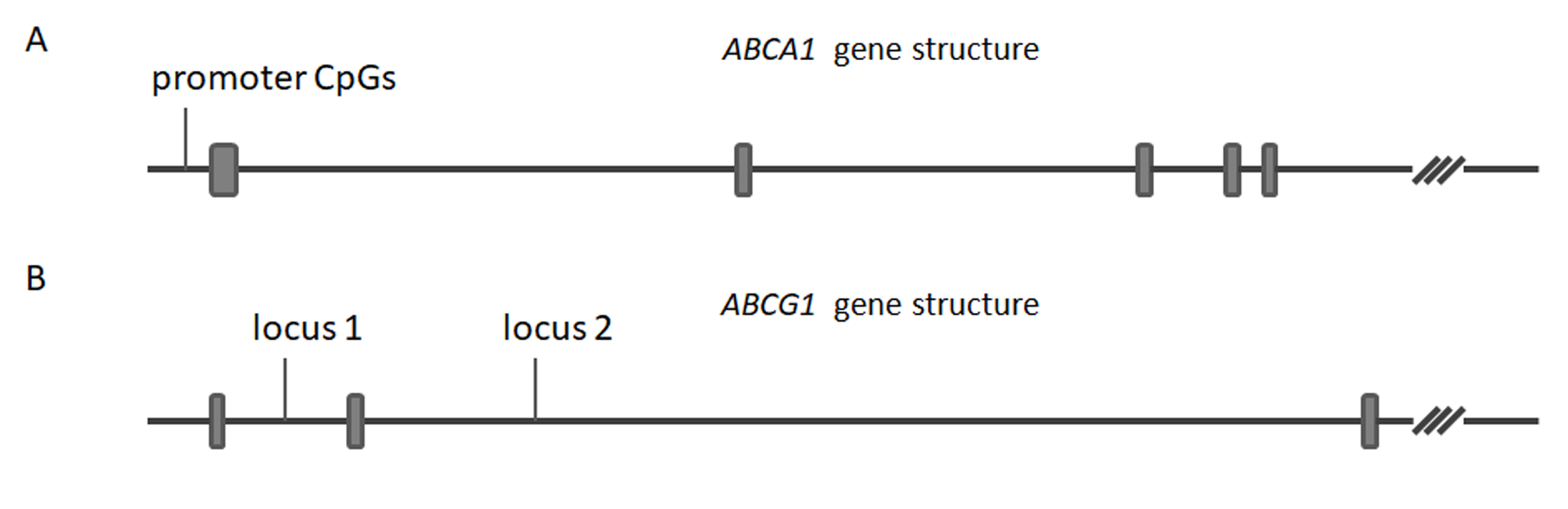

Supplement: Supplementary file 2 — Additional file 2: Fig. S1. Proportional diagram of the structure of ABCA1 (NM_005502.4) (A) and ABCG1 (NM_207174.1) (A) genes and loci selected for DNA methylation analysis. The line represents the gene (left to right: 5′–3′). Solid rectangles represent exons. Because of the long length of ABCA1 and ABCG1 genes, exons distant from the analyzed regions are omitted. ABCA1, ATP-binding cassette A1 gene; ABCG1, ATP-binding cassette G1 gene. [file 12872_2021_2379_MOESM2_ESM.png]

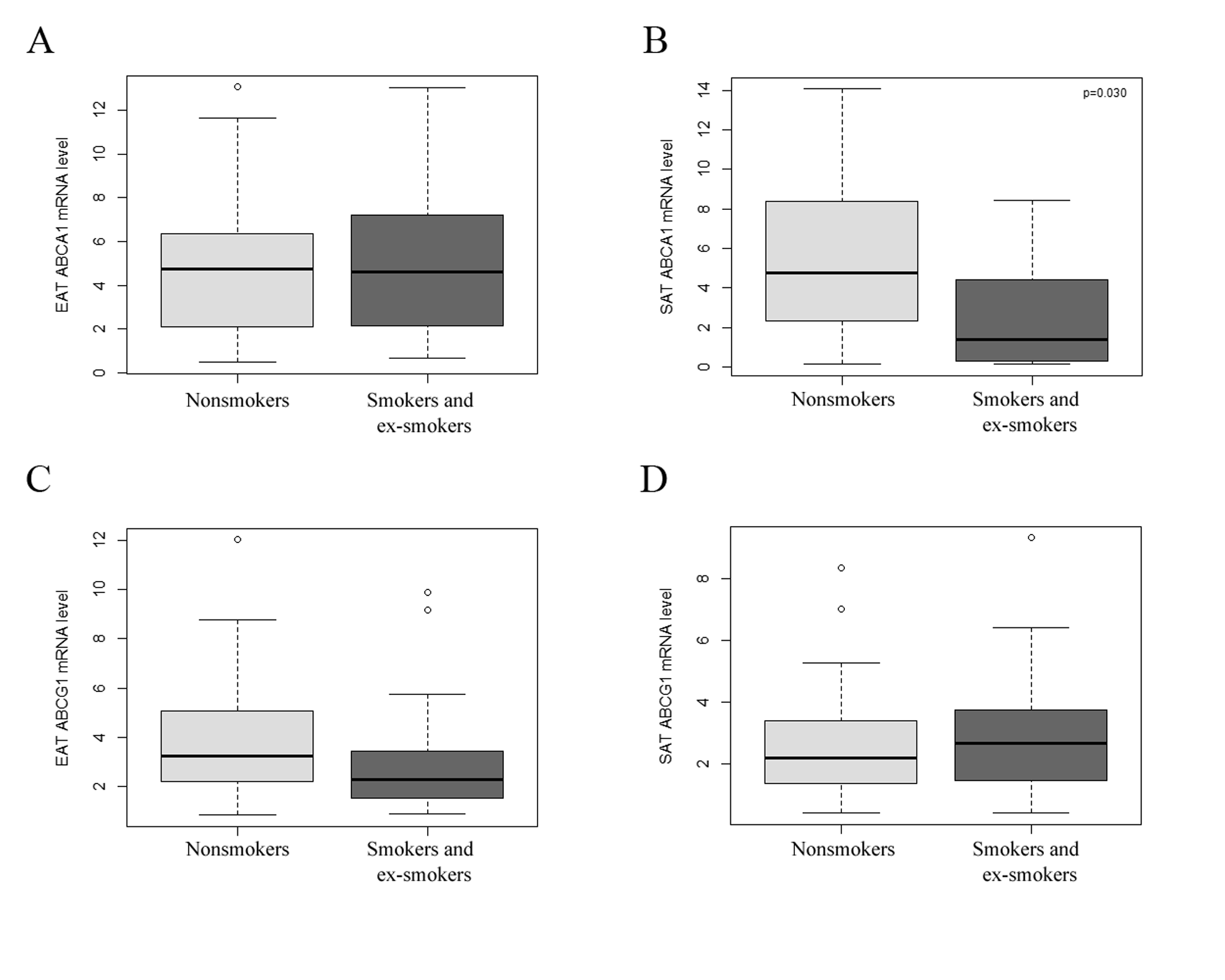

Supplement: Supplementary file 6 — Additional file 6: Fig. S2. ABCA1 and ABCG1 mRNA levels in EAT and SAT in subgroups divided according to smoking status. A ABCA1 mRNA levels in EAT; B ABCA1 mRNA levels in SAT (p = 0.030); C ABCG1 mRNA levels in EAT; D ABCG1 mRNA levels in SAT. [file 12872_2021_2379_MOESM6_ESM.tif]

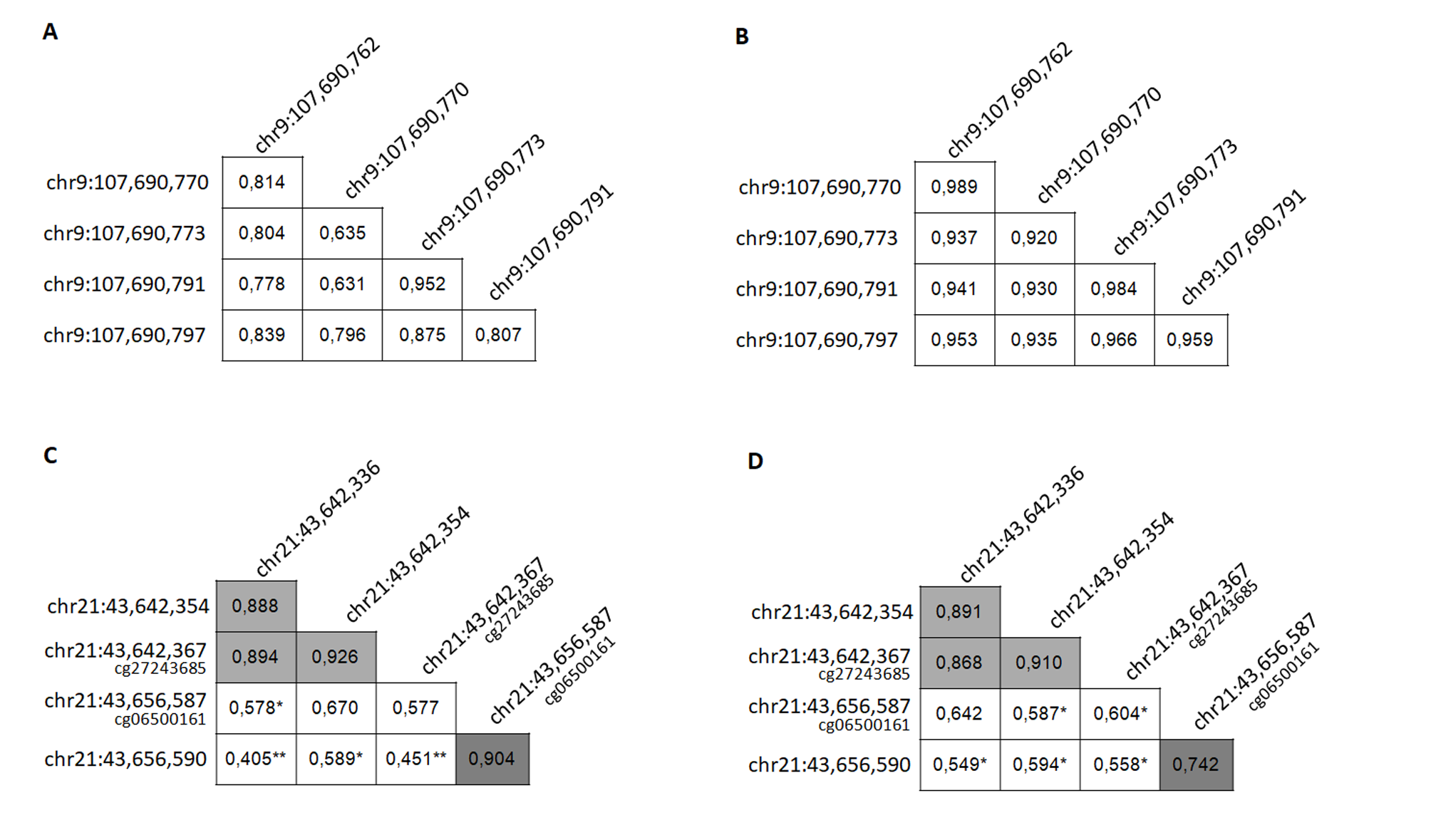

Supplement: Supplementary file 7 — Additional file 7: Fig. S3. Spearman’s correlation coefficients (r) between CpG site methylation levels within analyzed loci: A for ABCA1 in SAT; B for ABCA1 in EAT; C for ABCG1 in SAT; D for ABCG1 in EAT. Filled squares in the case of ABCG1 gene represent correlations in the same locus, unpainted—between two different loci. *p < 0,01; **p < 0,05; for rest cells p = 0.000. [file 12872_2021_2379_MOESM7_ESM.tif]
