# Supplementary material for: ABCA1 and ABCG1 DNA methylation in epicardial adipose tissue of patients with coronary artery disease
Source: BMC Cardiovasc Disord. 2021 Nov 27;21:566. doi: 10.1186/s12872-021-02379-7 (PMC8627066; doi:10.1186/s12872-021-02379-7)
Supplement: Supplementary file 3 — Additional file 3: Table S2. ABCA1 promoter DNA methylation levels (%) in EAT and SAT in studied groups. [file 12872_2021_2379_MOESM3_ESM.docx]

Table S2. *ABCA1* promoter DNA methylation levels (%) in EAT and SAT in studied groups.

| CpG dinucleotide position on the chromosome/  adipose tissue type | CAD  N=24(20) | NCAD  N=8(8) | p value  CAD vs NCAD |
| --- | --- | --- | --- |
| chr9:107,690,762  EAT  SAT | 18.51 (7.87-32.54)  8.20 (4.76-20.10) | 10.03 (5.36-17.85)  8.15 (4.15-11.99) | 0.015  >0,05 |
| chr9:107,690,770 (cg14019050)  EAT  SAT | 15.20 (4.56-25.99)  5.94 (3.54-13.75) | 6.89 (4.20-12.53)  6.20 (2.13-9.74) | 0.015  >0.05 |
| chr9:107,690,773  EAT  SAT | 28.68 (15.18-49.46)  14.74 (10.02-33.11) | 13.79 (10.02-25.58)  13.88 (6.77-24.07) | 0.004  >0,05 |
| chr9:107,690,791  EAT  SAT | 24.81 (12.12-40.73)  12.73 (8.51-27.62) | 11.17 (7.34-20.94)  10.84 (4.93-21.85) | 0.003  >0.05 |
| chr9:107,690,797  EAT  SAT | 22.38 (9.47-36.36)  11.14 (7.11-22.30) | 12.95 (7.94-20.93)  10.12 (6.13-15.42) | 0.012  >0.05 |
| Mean methylation level at analyzed locus (%)  EAT  SAT | 21.92 (10.29-36.93)  10.75 (7.15-22.52) | 10.81 (7.12-18.74)  9.59 (4.82-16.36) | 0.003  >0.05 |

Notes. N – EAT (SAT) samples analyzed.

p values were adjusted by the Holm-Bonferroni procedure.
