# Supplementary material for: ABCA1 and ABCG1 DNA methylation in epicardial adipose tissue of patients with coronary artery disease
Source: BMC Cardiovasc Disord. 2021 Nov 27;21:566. doi: 10.1186/s12872-021-02379-7 (PMC8627066; doi:10.1186/s12872-021-02379-7)
Supplement: Supplementary file 4 — Additional file 4: Table S3. ABCG1 DNA methylation levels (%) in EAT and SAT in studied groups. [file 12872_2021_2379_MOESM4_ESM.docx]

Table S3. *ABCG1* DNA methylation levels (%) in EAT and SAT in studied groups.

| Locus | CpG dinucleotide position on the chromosome/  adipose tissue type | NCAD  N=8(8)/9(9) | CAD  N=23(21)/22(19) | p value  CAD vs NCAD |
| --- | --- | --- | --- | --- |
| Locus 1 in Intron1 | chr21:43,642,336  EAT  SAT | 57.12 (41.91-67.76)  43.63 (37.17-52.36) | 66.75 (55.04-78.62)  49.77 (39.47-76.82) | 0.036  0.108 |
|  | chr21:43,642,354  EAT  SAT | 70.55 (53.22-76.15)  55.38 (48.77-64.55) | 74.83 (64.08-83.57)  61.49 (52.47-81.53) | 0.018  0.048 |
|  | chr21:43,642,367 (cg27243685)  EAT  SAT | 68.42 (51.56-70.88)  55.72 (45.18-67.57) | 71.51 (63.97-83.10)  61.38 (53.43-77.48) | 0.024  0.136 |
|  | Mean methylation level at analyzed locus (%)  EAT  SAT | 65.66 (49.39-71.60)  51.19 (45.36-60.41) | 71.15 (61.70-81.33)  57.55 (48.59-78.74) | 0.018  0.074 |
| Locus 2 in Intron2 | chr21:43,656,587 (cg06500161)  EAT  SAT | 37.79 (25.33-46.00)  31.68 (19.82-34.93) | 46.11 (33.04-59.76)  36.17 (25.87-68.55) | 0.016  0.048 |
|  | chr21:43,656,590  EAT  SAT | 48.54 (42.02-63.09)  48.42(36.29-51.75) | 60.86 (51.30-69.08)  52.41 (40.46-68.91) | 0.003  0.036 |
|  | Mean methylation level at analyzed locus (%)  EAT  SAT | 43.16 (33.78-54.32)  39.70 (28.06-42.31) | 53.57 (42.17-63.70)  43.65 (33.38-68.73) | 0.004  0.020 |

Notes. N – EAT (SAT) samples analyzed at locus 1/locus 2.

p values were adjusted by the Holm-Bonferroni procedure.
