# Supplementary material for: ABCA1 and ABCG1 DNA methylation in epicardial adipose tissue of patients with coronary artery disease
Source: BMC Cardiovasc Disord. 2021 Nov 27;21:566. doi: 10.1186/s12872-021-02379-7 (PMC8627066; doi:10.1186/s12872-021-02379-7)
Supplement: Supplementary file 5 — Additional file 5. The origin bands of WB.The origin bands of WB. [file 12872_2021_2379_MOESM5_ESM.docx]

***ABCA1* and *ABCG1* DNA methylation in epicardial adipose tissue of patients with coronary artery disease**

Valentina V. Miroshnikova^1,2^**^*^**, Alexandra A. Panteleeva^1,2,3^, Irina A. Pobozheva^1,2,3^, Natalia D. Razgildina^1^, Ekaterina A. Polyakova^2^, Anton V. Markov^4^, Olga D. Belyaeva^2^, Olga A. Berkovich^2^, Elena I. Baranova^2^, Maria S. Nazarenko^4^, Valery P. Puzyrev^4^, Sofya N. Pchelina^1,2,3^

^1^Petersburg Nuclear Physics Institute named by B.P. Konstantinov of National Research Centre “Kurchatov Institute”, Gatchina, Russian Federation

^2^Pavlov First Saint Petersburg State Medical University, St.-Petersburg, Russian Federation

^3^National Research Centre “Kurchatov Institute”, Moscow, Russia

^4^Laboratory of Population Genetics, Research Institute of Medical Genetics, Tomsk, Russian Federation

**^*^**for correspondence: [miroshnikova_vv@pnpi.nrcki.ru](mailto:miroshnikova_vv@pnpi.nrcki.ru)

**The origin bands of WB**

**
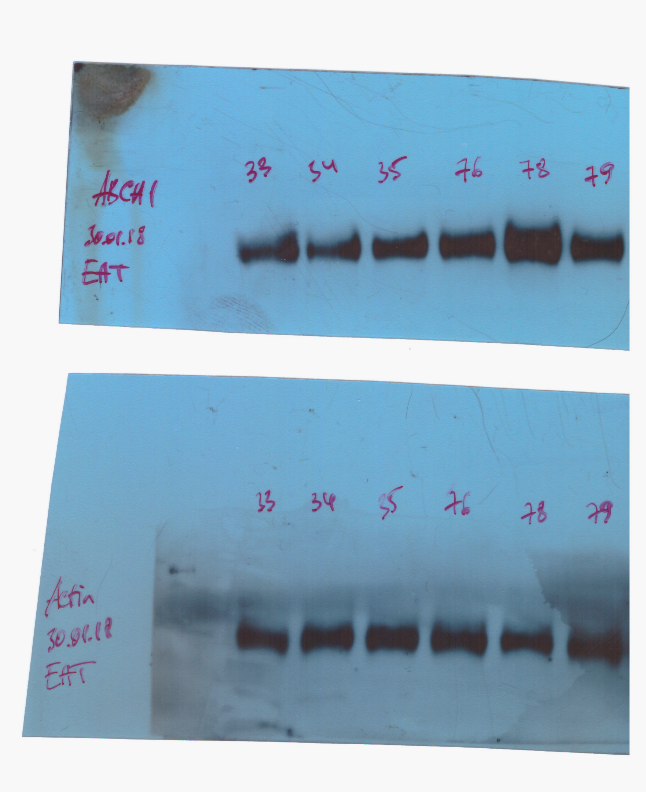
** CAD NCAD
